# The Influence of Ideological Variables in the Denial of Violence Against Women: The Role of Sexism and Social Dominance Orientation in the Spanish Context

**DOI:** 10.3390/ijerph17144934

**Published:** 2020-07-08

**Authors:** Jesús M. Canto, Macarena Vallejo-Martín, Fabiola Perles, Jesús San Martín

**Affiliations:** Department of Social Psychology, Social Work, Social Anthropology and East Asian Studies, Faculty of Psychology and Speech Therapy, University of Malaga, Campus Teatinos, 29071 Malaga, Spain; mvallejo@uma.es (M.V.-M.); fanovas@uma.es (F.P.); sangar@uma.es (J.S.M.)

**Keywords:** sexism, social dominance orientation, violence against women

## Abstract

Violence against women in heterosexual intimate relationships is a major social problem with serious physical and psychological consequences for the victims. There is a line of research that seeks to analyze how ideological variables and contextual variables influence the way in which this type of violence is perceived. This study analyzed the relationship between hostile sexism, benevolent sexism and social dominance in the acceptance of the myths about violence against women in intimate relationships. A total of 215 Spanish university students (125 women and 90 men) participated in the research. The results indicate that hostile sexism and social dominance orientation act as factors that influence the acceptance of such myths in men. Benevolent sexism did not act in this way. The data reconfirm the importance of hostile sexism and social dominance orientation in the perception of violence against women, in this case, that which is committed by their partners (or ex-partners) in the area of intimate relationships.

## 1. Introduction

It has been established that violence against women is transcultural, can be expressed in multiple ways, and causes grave physical and psychological harm to its victims [1]. The Declaration on the Elimination of Violence against Women defined the concept of violence against women as “any act of gender-based violence that results in, or is likely to result in, physical, sexual or psychological harm or suffering to women, including threats of such acts, coercion or arbitrary deprivation of liberty, whether occurring in public or in private life” [2] (p. 2). This definition includes a wide range of types of violence that women could suffer from, among which we can highlight physical violence, psychological violence and sexual violence by the partner (or ex-partner) or another family member; genital mutilation; rape; psychological abuse; harassment at the workplace; forced prostitution; etc.

One of the most common forms of violence against women is that which they experience in the area of intimate heterosexual relationships, known as IPVAW (Intimate Partner Violence Against Women). The UN has defined IPVAW as “any behavior within an intimate relationship that causes physical, psychological or sexual harm to those in the relationship, including acts of physical aggression (slapping, hitting, kicking and beating), psychological abuse (intimidation, constant belittling or humiliation), sexual coercion or aggression, or any type of controlling behaviors (isolating a person from family and friends, monitoring their movements and restricting access to information or medical care)” [3] (p. 89). Accordingly, this type of male violence refers to physical, psychological or sexual aggressions within the couple’s relationship that women suffer at the hands of the male partner. It is considered a public health problem because of the grave nature of its physical and psychological consequences [4], occurring in all countries and cultures [5], resulting in harmful effects for the victim as well as for the society as a whole [6]. At the global level, it is estimated that 30% of women have experienced physical and/or sexual violence committed by their partners at some moment in their lives [1], with women in the age range of 15 to 24 being most affected [7]. The seriousness of this type of violence is manifested by the fact that 38% of homicides of women worldwide are committed by partners or ex-partners [1]. Specifically, in Spain, according to the Ministry of Health, Social Services and Equality together with the Government Delegation on Gender Violence [8], 12.5% of the women participating in the survey reported having suffered physical or sexual violence at the hands of their partner or ex-partner. The private nature that IPVAW entails and its cultural acceptance could lead to its prevalence being underreported, so the number of women experiencing it could be greater. IPVAW is a phenomenon that has a high degree of causal complexity related to gender conceptions which have deep cultural roots, sustained by different ideological variables [9].

### 1.1. Social Perception of Violence Against Women by Their Male Partners in the Area of Intimate Partner Relationships

Social psychology has developed important research lines for analysis of the perception of different types of male violence committed against women [10,11,12,13]. The study of attitudes towards violence against women is a key element for understanding the use of this violence. According to the extent that a given sociocultural context accepts and justifies aggressive behavior towards women (in any of its forms), the likelihood increases for the aggressor engaging in this behavior as it does for the victims justifying the violent behavior of the aggressors and bystanders neither reporting such acts to the authorities nor supporting the female victims of the aggression [14]. The way in which bystanders perceive aggression against women influences the likelihood of dissuading the aggressor and of his social condemnation, the social downplaying of the negative consequences for victims and the social support provided to them, preventing them (or not) from being doubly victimized [15].

There is a whole series of factors involved in the distorted perception of IPVAW [12,16], characterized by questioning the credibility of the victim and by blaming her [17]. In spite of being considered a social problem and despite the provision of legislative, economic and educational means to combat this type of male violence, there is still a certain reticence to accept IPVAW as a reality in some sectors of society. Not only is there denial of its existence as a phenomenon but also reasons are found to justify the aggression (for example, previous insults, infidelity, going out without permission, etc.) along with blaming the victim for not putting a stop to such abuse and violent actions [18,19].

In the area of research into IPVAW perception or interpretation, studies have focused on the contextual characteristics of the situation of male violence, on the attitudinal and ideological variables of the observers as well as on the interaction between both these types of variables [12,16]. Among the contextual factors considered are the frequency of abuse [20], the type of relation between the aggressor and the victim [21], the type and severity of the aggression [22], and the previous reaction of the victim [23], among others. From the set of ideological attitudes that are involved in the perception and interpretation of acts of IPVAW, a variable has been proposed on a specific trait involved in the perception of IPVAW called “myths about IPVAW” [11,16,24]. The perception of IPVAW is built on myths, stereotypes and attitudes about the victims themselves, the aggressors and the types of intimate partner relationships considered appropriate in the society. Megías et al. define these myths as “descriptive or prescriptive beliefs about rape (i.e., about its causes, contexts, consequences, perpetrators, victims and their interaction) that serve to deny, downplay or justify sexual violence that men commit against women” [16] (p. 47). Taking as an antecedent the research on rape myth acceptance [10,25], these myths are taken as beliefs of a cultural nature that reinforce violence, thwarting its detection and downplaying the existence of violence in intimate partner relationships by men, blaming the victim, exonerating the aggressor and encouraging proclivity to act violently [26].

Bosch and Ferrer [11,27] carried out an analysis of myths about IPVAW aimed at classifying them according to their importance, stressing the existence of such myths (myths about IPVAW: marginality, a phenomenon that only occurs among the lowest social classes; myths about abusers: they have mental problems; myths about abused women: the women provoke the aggression and/or have a masochistic and submissive nature; and myths that downplay the importance of gender violence: it is a type of violence that is not common and has scant consequences for the victim), all of them having the specific function of denying IPVAW. For those denying IPVAW, they view it as only representing a small minority and as a result of interpersonal discrepancies between the couple within the private domain [28].

The interpretation of any act of male violence against women in the area of relationships also depends on other attitudinal and ideological variables of the observer. These variables influence the degree of blame attributed to the victim and to the aggressor, and they are influenced by the acceptance of traditional gender stereotypes [22] and by the acceptance of sexist attitudes [29,30]. The sexist attitudes are exacerbated when the observers think that the women do not fit into the traditional stereotyped roles as mothers and wives, according to which they blame more those female victims of aggression when then do not fulfill expectations dictated by these traditional roles [20]. 

Framed into the patriarchal system of discrimination against women, sexism is an ideological variable that influences the interpretation of the aggressions toward women [31]. It is a construct close in content to the myths about women’s acceptance of violence [32] as well as myths about IPVAW. By sexism, we understand a gender ideology that implies a set of beliefs about the roles considered appropriate for men and women as well as about the relationships that the members of both groups should maintain [33]. Glick and Fiske [34] affirm that one of the characteristics of today’s Western societies is the coexistence of a sexist antipathy toward women and alongside positive feelings towards them. This type of sexism, which is termed ambivalent sexism, distinguishes between two closely linked components: hostile sexism (HS) and benevolent sexism (BS). According to these researchers, individuals scoring high in HS assume that women are weaker than men and are not competent to govern social institutions and believe that females are dangerous and manipulators due to the sexual power they exert over men. BS, on the other hand, implies that men assume a role of protecting women and that the latter have positive characteristics which are complementary to men and assume that there is a dyadic dependence by men with respect to women. If HS is characterized by clear antipathy and rejection of women, above all toward those challenging the established power, BS assumes that women are better at determined roles (e.g., mother and wife) because they are weaker than men. Benevolent sexists have a positive image of those women who fit into the submissive role, but they reject, as do hostile sexists, those that challenge the established order. HS serves to safeguard the status quo, punishing women who deviate from traditional gender roles, while BS offers protection to women in exchange for complacency in their own subjugation [35].

HS and BS serve to maintain and justify the structural power of men, correlating both positively [36]. It was found that individuals who assume sexist attitudes accept the use of violence in intimate relationships to a greater degree [37]. Sexism negatively affects the image of the victims in the aggressions and beliefs about the causes of these aggressions [38]. Valor-Segura et al. [30] affirm that HS legitimizes male violence against women who challenge men’s power, who “take advantage” of men sexually, and who tarnish their honor. HS perceives the man as a victim of the woman’s sexual power, subject to possible manipulations of a sexual nature that could lead the man to aggression in response to the woman’s offense. Thus, HS fits in with one of the myths about IPVAW in being in agreement with the supposition that it is the woman who provokes her partner’s aggressions towards her. Accordingly, it can be expected that individuals scoring high in HS deny that violence against women exists in the area of partner intimate relationships as a structural fact of domination and would be more in agreement with the myths on IPVAW being a way to deny gender violence. At the same time, BS holds a stereotyped view of women as fragile beings who carry out traditional feminine roles and whose attitudes lead them to being perceived as a complement to a man. This generates positive feelings towards women, including protection against possible aggressions from other men. As such, we cannot expect BS to predict acceptance of myths about IPVAW.

Furthermore, some studies have paid attention to the relationship between the perception of violence against women and ideological variables that are defined as antecedents of prejudice [32,39,40], as social dominance orientation (SDO) [41]. This variable acts as an antecedent towards a large number of outgroups and can likewise explain the links to perception of violence toward women. Some research has pointed to the existence of a relationship between justification of sexual violence toward women and SDO [32,42,43]. According to the theory of social dominance, it is assumed to be an ideological variable that produces a general position towards intergroup relations. People who obtain high scores in SDO defend policies that reinforce the hierarchies between groups and individuals, and those people prefer the superiority of their ingroup over outgroups [41]. In accordance with this theory, these authors maintain that the different forms of oppression present in intergroup relationship (racism, sexism, classism, etc.) are specific cases of a more general tendency of human beings to form and maintain hierarchies on the basis of groups. What SDO would reflect would be the trend of human beings classifying social groups through the superiority–inferiority dimension and to favor policies that maintain social inequality. SDO is an individual variable that focuses on the general tendency in favor of myths and stories that legitimize the expansion of the hierarchy and support group inequalities in opposition to egalitarian postulates. Diverse studies have obtained positive correlations between SDO and conservative beliefs, such as ethnic prejudices, economic and political conservatism, and a preference for right-wing parties [44]. Defending hierarchical relationships causes high scorers in SDO to denigrate members in particular members of the outgroup that support equal rights (for example, feminists, organized ethnic minorities, groups which defend homosexuals, etc.) to reinforce their own status. Stereotypes play the role of legitimizing myths that people high in SDO use as a means of justifying their negative attitudes [41]. SDO is rooted in a need to maintain social group hierarchy. SDO is closely related to hostile sexism [45] and is associated with the maintenance and defense of traditional values, hierarchies and gender relations typical of more conventional, patriarchal societies [46]. Following the approach of Süssenbach and Bohner [32] applied to myths about rape, it can be assumed that violence against women in intimate relationships and the myths denying this male violence are a form of domination over women of which the purpose is to maintain the existing power hierarchy in which the man dominates the women. Individuals who score high in SDO would accept to a greater degree the dominance of the man over the woman and assumptions of myths about IPVAW as a form of domination. The close link between SDO and SH, conceiving SH as an expression of the need to maintain the group hierarchy that places the man in a position of superiority with respect to the woman, can lead us to conclude that individuals scoring high in both variables are those who accept the myths about IPVAW to a greater degree. Individuals scoring high in both variables, upon accepting, to a greater degree, myths regarding IPVAW, not only deny violence committed against women but also would deny the existence of male dominance over the female.

### 1.2. Objectives and Hypothesis

This study has focused on the influence of ideological variables (HS, BS and SDO) on the myths about male violence against women in intimate relationships. In this study, we seek to determine whether HS, BS and SDO influence the degree of acceptance of the myths about IPVAW in a Spanish sample. The study carried out by Glick et al. [37] obtained results that situate Spain at a medium level of sexism (higher than the countries of northern Europe but lower than the countries of South America). With regard to this study’s objectives, we formulated the following hypotheses:

**Hypothesis** **1:**  *It was expected that those participants who scored higher in HS would accept myths about IPVAW to a greater degree*.

**Hypothesis** **2:**  *It was not expected that those participants who scored higher in BS would accept myths about IPVAW to a greater degree*.

**Hypothesis** **3:**  *It was expected that those participants who scored higher in SDO would accept myths about IPVAW to a greater degree*.

**Hypothesis** **4:**  *It was expected that those participants who scored higher in SH and SDO would accept myths about IPVAW to a greater degree*.

## 2. Materials and Methods 

### 2.1. Participants and Procedure

Two hundred and fifteen people voluntarily participated in this study (90 men and 125 women), ranging in age from 18 to 33. The average age was 21.62 (*SD* = 4.16). The participants were students at Málaga University (Spain), Faculty of Psychology and Speech Therapy. A priori analysis with G*Power software (3.1.9.7 version, University of Düsseldorf: Düsseldorf, 2020) estimated a sample of 195 as adequate for this design with 80% power to detect a moderate effect among the variables (*f* = 0.25).

The questionnaires were answered collectively and anonymously in the students’ classes for approximately 25 min. The researchers instructed them to respond individually to all the items. They were informed that their answers would be completely anonymous and were guaranteed absolute confidentiality in the handling of the information. In addition, participants read a debriefing explaining the goals of the study and they also had the opportunity to request an additional oral debriefing. The ethical guidelines of this research were approved by the Research Ethics Committee of the University of Málaga.

### 2.2. Instruments

A booklet was prepared that included the following questionnaires:

Sociodemographic data: On the first page, the participants had to indicate their gender, age, and the degree program they were.

Acceptance of Myths about Intimate Partner Violence Against Women Scale (AMIVAW) in its Spanish version [16] assesses acceptance of myths about IPVAW through 15 items grouped into five sets of items to evaluate each of the myths: myths that excuse the aggressor, myths that blame the victim, myths that downplay the problem, myths that involve sociocultural factors, and myths that involve social and legal responses to the problem. This is a Likert-type scale with a range of responses from (1) strongly disagree to (7) strongly agree. A Cronbach’s Alpha = 0.91 was obtained.

Inventory of Ambivalent Sexism [33]: This inventory was translated into a Spanish version [47] to assess ambivalent sexism. It is composed of 22 items (11 corresponding to hostile sexism and 11 corresponding to benevolent sexism), with a Likert-type scale format (1 = strongly disagree; 7 = strongly agree). Cronbach’s Alpha in SH and in BS were obtained (0.83 and 0.85 respectively). 

Social Dominance Orientation (SDO): We used the Spanish version [48] to assess SDO. This scale consists of 16 items (Likert-type, 1 = strongly disagree; 7 = strongly agree). Higher scores indicate higher levels of SDO. A Cronbach’s Alpha = 0.85 was obtained. 

## 3. Results

SPSS (version 24.0, SPSS Inc., Chicago, IL, USA, 2016) was used to carry out the statistical analyzes. An initial exploratory analysis of the data was carried out to detect potential outliers. They were not detected so it was not necessary to reduce the initial sample. To check differences according to the participant’s gender in AMIVAW, HS, BS and SDO, Student’s t-test was carried out.

A Pearson’s correlation analysis was conducted between AMIVAW, SH, BS and SDO. Table 1 presents descriptive statistics.

Significant correlations were found between all the variables of the study. AMIVAW correlated in a high and significant way with HS (*r* = 0.745; *p* < 0.001) and in a moderated and significant way with BS (*r* = 0.403; *p* < 0.001) and with SDO (*r* = 0.435; *p* < 0.001). HS correlated in a high and significant way with BS (*r* = 0.610; *p* < 0.001) and in a moderated and significant way with SDO (*r* = 0.495; *p* < 0.001). BS correlated in a moderated and significant way with SDO (*r* = 0.349; *p* < 0.001).

Looking at the scores obtained according to gender, the results of the t-test showed no significant differences in BS (*Mmen* = 2.43; *SD* = 0.83; *Mwomen* = 1.99; *SD* = 0.71; *t* = 1.15; *p* = 0.105). Significant differences were obtained in AMIVAW (*Mmen* = 2.78; *SD* = 0.85; *Mwomen* = 2.10; *SD* = 0.74; *t* = 5.03; *p* = 0.001; *d* = 1.15), HS (*Mmen* = 2.59; *SD* = 1.00; *Mwomen* = 1.79; *SD* = 0.75; *t* = 5.87; *p* = 0.001; *d* = 1.13), and SDO (*Mmen* = 2.57; *SD* = 0.86; *Mwomen* = 2.26; *SD* = 0.77; *t* = 2.25; *p* = 0.026; *d* = 0.31).

A multiple regression analysis was carried out using sex, HS, BS and SDO as predictor variables and AMIVAW as a criterion variable (*R2c* = 0.580; *F* = 46.02, *p* = 0.001). In step 1 of the regression analysis, the sex of the participants was introduced as a control variable. In step 2, three variables were included (standardized HS, BS and SDO) and interaction between SH with SDO in step 3 (moderation analyses). This was done to verify whether SH affected the criterion variable differently depending on SDO (Table 1).

In step 1, a significant effect of sex on AMIVAW was found (*β* = 0.367, *t* = 5.05, *p* = 0.001), so that men scored much higher than women for this variable. In step 2, we found two significant main effects on AMIVAW. Participants scored higher in acceptance of AMVBAW if they (a) were high (rather than low) in HS (*β* = 0.572, t = 7.92, *p* = 0.001) and if they (b) were high (rather than low) in SDO (*β* = 0.139, *t* = 2.43, *p* = 0.016). Participants did not vary based on their score in BS (*β* = 0.105, *t* = 1.54, *p* = 0.124). There was no interaction between SH and SDO (*β* = 0.019, t = 0.33, *p* = 0.736; Table 2).

## 4. Discussion

The aim of this research was to analyze the role played by HS, BS and SDO in acceptance of myths about male violence against women in intimate partner relationships in a Spanish context. As was formulated in Hypothesis 1, participants with higher levels of SH accepted, to a greater degree, myths about IPVAW, which implied denying the existence of violence against women in intimate partner relationships as something structural and, as such, considering it as a lesser type of violence. These myths involve accepting this violence as a marginal phenomenon pertaining to lower social classes, typical of men with serious psychopathological problems and of women who provoke aggression and/or with certain masochistic tendencies. This would result in violence against women being considered an isolated phenomenon and with scant physical or psychological consequences for the victims [11]. What people that accept these myths do is deny the existence of male violence against women in intimate relationships as gender violence, covering up the fact that male aggression against women is aimed at control and dominance by the man.

In this research, it has once again been shown that sexism is an ideological variable that influences interpretations of aggressions against women [31,38]. Sexism negatively affects the image of the female victims of male violence, underestimates the frequency of the aggressive actions and distorts the causes attributed to these aggressions. In the theoretic models explaining violence against women, such as the model proposed by Bosch et al. [27] based on the patriarchal substrate, it places men in a privileged hierarchical position with respect to women. This is imprinted in individuals through the socialization process, leading to a gender differentiation process sustained by sexist attitudes and beliefs that prescribe certain behaviors for men and others for women, sexist attitudes that represent expectations for control in women assumed not only by men but also by the women themselves.

The hostile dimension of ambivalent sexism, characterized by clearly reflecting an obvious antipathy and rejection towards women who challenge the established power and which legitimizes violence against women in intimate relationships labeled as manipulators for using their sexual power to “harm” the man [33,34,37], predicts myths about IPVAW by being in agreement with the supposition that it is women who provoke their partner’s aggression. Thus, on one hand, HS justifies the man’s sporadic aggression against a woman, perceiving it as an “act of defense”, but on the other hand, accepting the myths about IPVAW, they deny that violence against women is a generalized phenomenon. Accordingly, upon denying it, the individuals who score high in SH must believe that many of the domestic violence complaints filed by women in the area of intimate relationships are false. HS opposes equalitarian ideology and correlates negatively with feminist demands, one of which is implementation of more effective measures to eradicate male violence against women [13].

The data has confirmed Hypothesis 2. Participants’ adherence to BS has not been linked to a greater acceptance of myths about IPVAW. BS has not constituted a predictor of myths about IPVAW. Individuals scoring high in BS have a positive image of women who fit into the submissive role and expect and defend a man’s role as protector with respect to women. The benevolent dimension of sexism does not show a hostile attitude toward women but rather expresses positive feelings towards them when they fit into the traditional canons derived from the BS stereotypes. The use of sexist attitudes implied in HS as well as BS, as reflected in the positive correlations between both variables, lead both types of sexism to be used in a differential way with distinct types of women: BS for submissive women and HS for women that defy the established order [33,35]. Accordingly, BS serves to mask the relationship of dominance by providing a certain type of recognition for those women who carry out traditional roles. This type of sexism does not act as discourse that reflects hostility toward women. Instead, it acts as a discourse of protection against the possible aggressions that women could suffer, while at the same time, it is ideologically based on HS and directs hostility toward those women that challenge the established order.

SDO predicted acceptance of myths about IPVAW (Hypothesis 3). This implies that accepting the postulates of SDO is in itself sufficient for participants to accept, to a greater degree, myths about IPVAW. Persons who score high in SDO would accept men’s dominance over women to a greater degree and assume myths about IPVAW as a form of dominance. Myths that deny violence against women are a form of male dominance aimed at maintaining the existing power hierarchies in which men dominate women. Individuals scoring higher in SDO accept intergroup relations based on inequality to a greater degree. SDO is rooted in a need to maintain social group hierarchy and is closely related to hostile sexism [45]. The data obtained in this study has shown a higher positive correlation between SDO and HS than between SDO and BS. HS places the woman in a lower tier compared to men, so that HS can act as an ideological variable to justify the relationship of inequality defended by persons who score high in HS. The inferiority attributed to women justifies the fact that relationships between men and women are not equitable and justifies the relationship of dominance, a relationship that is concealed behind sexist attitudes toward women. Thus, acceptance of the SDO postulates predicts denying that violence against women is of a structural order, accepting to a greater degree the myths about IPVAW.

The data did not confirm that HS moderates the link between SDO and myths about IPVAW (Hypothesis 4). It was expected that those individuals with higher scores in HS and SDO would be those scoring higher in acceptance of myths about IPVAW. These individuals show greater hostility toward women and are those who are more in favor of inequality. The influence of HS in the score obtained in myths about IPVAW does not vary according to the scores obtained in HS by the participants.

The scores obtained in acceptance of myths about IPVAW, measured through AMIVAW, were not high. Young Spanish university students usually show such a pattern in the results [43]. This study found that men, in comparison with women, assumed more myths about violence against women and scored higher in HS and in SDO. In all of these variables, the scores were low. The high correlation between AMIVAW and HS, BS and SDO imply that denial of structural violence against women is sustained by a series of attitudes of an ideological nature. This represents a sociocultural risk factor in the origin of the violence itself and in maintaining it [3]. Together with the role carried out by variables close in content to acceptance of myths about violence against women such as HS and BS, the present study reveals that SDO also serves as an ideological variable that sustains acceptance of such myths as a tool to control women by denying the very existence of this violence, even when it is exercised against women.

### Limitations and Future Research

Future research should select samples of the population from distinct social classes and different cultural backgrounds and not solely individuals from a university ambit in order to analyze to what degree the scores obtained in myths about IPVAW, HS, BS and SDO are higher than those obtained in this study and examine how these variables are linked with populations that are more prone to discrimination against women and with ideologies where sexist and conservative attitudes predominate. In addition, it should include other ideological variables to attempt to analyze the structure on which such beliefs are constructed that sustain myths denying violence against women by their partners or ex=partners. The women that experience violence, those observing it and the men who commit it must be made aware of the ideological framework sustaining beliefs of a sexist nature regarding the behavior assigned to women and men and thus, from the perspective of prevention, be able begin deconstructing myths that shape the roles associated with masculinity and femininity. 

This study has found that men accept myths about IPVAW to a greater degree than do women. We consider it necessary for future research to analyze the role played not only by sex but also by gender as a social construct [49]. The social changes that include equalitarian measures and increased rights for women have brought about new ways to redefine femininity and masculinity. This has led to a certain gender insecurity in men that can increase hostility towards women and, as such, increase acceptance of aggressive acts by men against women [50].

The perception and interpretation of acts of violence against a woman by her partner or ex-partner depend on attitudinal variables, contextual variables and the interaction of both these types of variables. Future research should analyze how myths about IPVAW influence the interaction with attitudinal as well as with contextual variables in the way in which observers blame the aggressor and the victim. The research presented here reveals some of the underlying attitudinal variables in the consolidation of myths about IPVAW, such as ambivalent sexism and SDO.

## 5. Conclusions

Important lines of research have analysed the influence of ideological variables on the perception and interpretation of violence against women [10,11,12]. These studies have pointed out that a large part of the population sustains and admits myths that deny this type of violence. In the specific case of myths about IPVAW, the people who most accept these myths deny that violence against women in intimate relationships is structural in nature and define it as a primarily minority type of violence unrelated to male domination [12,16]. In this study, we showed that the higher the scores on HS and SDO, the greater degree of acceptance of the myths of IPVAW. People who score high on each of those variables by assuming the myths about IPVAW would deny the very existence of IPVAW as a widespread phenomenon. Specifically, HS is the type of sexism that predicts myths about IPVAW by expressing a hostile attitude towards certain types of women who are seen as sexually manipulative and who provoke male aggression in the context of intimate relationships. This perception towards women would justify the aggressions carried out by men who are victims of female manipulation, aggressions that are considered to be rare and of a marginal nature. On the other hand, SDO also predicted the acceptance of the myths about IPVAW. SDO is sensitive to groups that challenge intergroup hierarchical relations. Those who score high on ODS would more likely embrace male domination and would take on more of the myths about IPVAW as a discourse justifying male domination over women. Such a discourse would help to maintain this domination by denying the existence of a violence against women in which the function is to control and dominate them.

## Figures and Tables

**Table 1:** Descriptive statistics and Pearson correlation values between variables.

	Mean	SD	AMIVAW	HS	BS
AMIVAW	2.44	0.80			
HS	2.19	0.87	0.745 *		
BS	2.21	0.75	0.403 *	0.610 *	
SDO	2.58	0.81	0.435 *	0.495 *	0.349 *

*p < 0.001. Note: AMIVAW, Acceptance of Myths about Intimate Partner Violence Against Women Scale; HS, Hostile Sexism; BS, Benevolent Sexism; SDO, Social Dominance Orientation.

**Table 2:** Multiple regression analysis on myths of IPVAW (Interpersonal violence against women)

		*β*	*t*	*p*
Step 1				
	Sex	0.367	5.05	0.001
Step 2				
	HS	0.572	7.97	0.001
	BS	0.105	1.54	0.124
	SDO	0.139	2.43	0.016
Step 3	Δ R^2^c = 0.372			
	SH*DO	0.019	0.23	0.736
	Δ R^2^c = 0.001			

Notes: HS, Hostile Sexism; BS, Benevolent Sexism; SDO, Social Dominance Orientation.
